# Assessing Mortality Disparities Among Non-Alcoholic Fatty Liver Disease Metabolic Dysfunction Fatty Liver Disease and Metabolic Dysfunction-Associated Liver Disease: A Comprehensive Meta-Analysis

**DOI:** 10.7759/cureus.71639

**Published:** 2024-10-16

**Authors:** Fahad Lakhdhir, Agha Syed Muhammad, Ahmed Nasir Qureshi, Imran A Shaikh, Imran Joher, Jawaria Majeed, Javaria Khan

**Affiliations:** 1 Adult Cardiology, National Institute of Cardiovascular Diseases, Karachi, PAK; 2 Gastroenterology, Hayatabad Medical Complex, Peshawar, PAK; 3 Gastroenterology, Russells Hall Hospital, Dudley, GBR; 4 Surgery, Ghurki Trust Teaching Hospital, Lahore, PAK; 5 College of Medicine, Liaquat University of Medical and Health Sciences (LUMHS), Hyderabad, PAK; 6 Medicine, Shalamar Medical and Dental College (SMDC), Lahore, PAK; 7 Gastroenterology and Hepatology, Nishtar Medical University, Lahore, PAK; 8 Internal Medicine, Jinnah Hospital, Lahore, PAK

**Keywords:** cardiovascular disease, cirrhosis, fibrosis, meta-analysis, metabolic dysfunction-associated fatty liver disease, mortality, non-alcoholic fatty liver disease, nonalcoholic steatohepatitis

## Abstract

Non-alcoholic fatty liver disease (NAFLD) has emerged as a major global health concern due to its association with increased mortality. While previous studies have indicated a link between NAFLD and mortality, variations in risk factors such as age, sex, and disease severity warrant a comprehensive meta-analysis to clarify these associations. This meta-analysis aimed to evaluate the overall cardiovascular disease (CVD) mortality risk associated with NAFLD, considering various subgroups defined by age, sex, disease severity, presence of cirrhosis or fibrosis, study quality, and follow-up duration. A systematic search of eight studies was conducted to assess the hazard ratios (HRs) for all-cause and CVD mortality associated with NAFLD. Heterogeneity among studies was evaluated using the I² statistic, and subgroup analyses were performed based on participant age, sex, disease severity, presence of cirrhosis or fibrosis, study quality, and follow-up duration. NAFLD was significantly associated with an increased risk of all-cause mortality (HR = 1.34, 95% CI: 1.17-1.54, I² = 80.0%). Subgroup analyses revealed that individuals aged ≥50 years (HR = 1.50, 95% CI: 1.31-1.73) and males (HR = 1.43, 95% CI: 1.29-1.59) had a higher mortality risk. Patients with nonalcoholic steatohepatitis (NASH) showed a significant association with both overall (HR = 1.37, 95% CI: 1.14-1.65) and CVD mortality (HR = 1.56, 95% CI: 1.25-1.97). The presence of cirrhosis or fibrosis further elevated the risk of mortality (HR = 3.22, 95% CI: 2.40-4.33). However, NAFLD was not significantly associated with CVD mortality in the overall analysis (HR = 1.13, 95% CI: 0.92-1.38). Heterogeneity was high across most subgroups, indicating varying effects based on study characteristics. NAFLD is significantly associated with increased all-cause mortality, particularly in older individuals, males, and those with NASH or cirrhosis/fibrosis. The association with CVD mortality was not significant in the overall analysis but was evident in specific subgroups. These findings underscore the importance of early detection and tailored management of NAFLD to mitigate its impact on mortality. Further research is needed to elucidate the mechanisms linking NAFLD with adverse health outcomes and to develop effective interventions.

## Introduction and background

The most prevalent form of chronic liver disease, non-alcoholic fatty liver disease (NAFLD), affects 22% to 33% of people worldwide [1]. NAFLD is a broad term that includes a variety of conditions, from non-alcoholic steatohepatitis (NASH) to non-alcoholic fatty liver [2-4]. It is well-recognized that having NAFLD increases the risk of cardiovascular disease and death [2,3]. Particularly in the latter stages, an increase in the fibrosis stage is linked to liver-related outcomes such as hepatocellular carcinoma [5-7], decompensation, and overall mortality [8]. Increased portal vascular resistance brought on by the changes in the hepatic architecture in fibrosis elicits portal hypertension, which is mechanistically linked to hepatic decompensation [9,10].

A more recent term, metabolic dysfunction-associated fatty liver disease (MAFLD), was used to highlight the metabolic foundations of fatty liver disease. As opposed to NAFLD, which is diagnosed by ruling out alcoholism or other liver disorders, MAFLD is identified by metabolic dysfunction [11]. The global prevalence of metabolic dysfunction-associated fatty liver disease, or MAFLD, is sharply increasing in tandem with the global obesity pandemic [12,13]. As of right now, this increasingly common liver disease affects about one in three people on the planet [14]. Due to the increased use of medical resources, this results in a significant healthcare burden and rising healthcare expenses [15]. About 25% of patients with MAFLD go on to develop metabolic dysfunction-associated steatohepatitis (MASH), a more severe form of the disease that includes inflammation-induced hepatocyte damage, hepatocyte ballooning, the development of liver fibrosis, and ultimately end-stage liver disease requiring liver transplantation. In the US, MAFLD is increasingly being listed as a reason for liver transplantation [15]. 

Metabolic dysfunction-associated liver disease (MDALD) is less commonly referenced and is used to describe liver disease associated with metabolic dysfunction, not limited to fatty liver [16]. It can include various liver conditions where metabolic factors are a central component. While there isn't a widely accepted separate diagnostic criteria set for MDALD, it generally includes liver diseases with metabolic dysfunction at their core. This term might encompass a broader range of liver conditions beyond simple steatosis, such as liver fibrosis and cirrhosis in the context of metabolic dysfunction [17].

The term non-alcoholic fatty liver disease (NAFLD) was used to describe MAFLD until recently. In addition to reflecting something it was not, the previous "exclusionary" name (non-alcoholic) was also thought to be stigmatizing. Consequently, a more encompassing word, MAFLD, was selected by Eslam et al. as part of a nomenclature reform proposal [11]. Although alcohol usage was seen as potentially problematic, this new phrase did not preclude it, and the term "fatty" was still thought to contribute to stigma [18]. Thus, "Steatotic Liver Disease (SLD)," a broad name, was approved in a recent multi-round, multi-stakeholder Delphi process [19]. Several particular reasons are defined under the general heading of SLD, such as MASH (metabolic dysfunction coupled with steatotic liver disease) and MetALD (metabolic dysfunction mixed with alcoholic liver disease). A deeper comprehension of the metabolic variables causing liver disease is reflected in the changing definitions of NAFLD, MAFLD, and MDALD [20]. It's still unclear, though, how these differences affect death rates. A thorough assessment of the differences in mortality between metabolic dysfunction and liver disease is necessary, considering the increasing frequency of these illnesses worldwide. In order to systematically assess cardiovascular, liver-related, and all-cause mortality amongst NAFLD, MAFLD, and MDALD populations, this meta-analysis was conducted.

## Review

Methods

Search Strategy

A search for publications up to 30^th^ July 2024 was done in the PubMed library databases using the Preferred Reporting Items for Meta-Analyses (PRISMA) guidelines [21]. From 2019 to 2024, the search terms "NAFLD," "MAFLD," "MDALD," "mortality," "liver disease," "epidemiology," and metabolic dysfunction were used. The search approach was modified based on a previously published systematic review of NAFLD. Thirteen were used to get rid of duplication, and all references were imported into Endnote X9. For a thorough search, manual screening of the references in the included articles was also carried out.

Eligibility and Selection Criteria

Before a full-text evaluation of the chosen papers, the titles and abstracts were checked for eligibility. Conflicts were settled, and research that met the following requirements was taken into consideration for inclusion: (3) adult individuals with NAFLD, MAFLD, and MDALD over the age of eighteen; (1) epidemiological studies (reporting mortality); and (2) biopsy-confirmed diagnosis of NAFLD, MAFLD, and MDALD. Time-dependent data and cross-sectional research must be removed from the meta-analysis according to its concept. Articles in the English language alone were considered for inclusion, and no date filter was used. Commentaries, editorials, systematic reviews, meta-analyses, case control, and conference abstracts were not included. Furthermore, research conducted in the pediatric population as well as studies reporting liver-related events without mortality data were excluded. In cases where a single cohort yielded numerous studies, only the most recent cohort was analyzed.

Data Extraction and Outcomes

From the included articles, pertinent information was extracted with a focus on three main areas: (1) study characteristics (year, country, study design); (2) patient characteristics (total sample size, age, gender, comorbidities like diabetes or hypertension; sample sizes for NAFLD, MAFLD, and MDALD); and (3) outcomes (all-cause mortality, liver-related mortality). Differences were settled. All-cause mortality in patients with NAFLD, MAFLD, and MDALD at various fibrosis levels was the main result of this meta-analysis. Liver-related death in these groups at varying degrees of fibrosis was the secondary outcome. Deaths from hepatocellular carcinoma, liver cirrhosis, chronic liver disease sequelae, or hepatic failure were classified as liver-related mortality.

Quality assessment

The quality of the included studies was evaluated using a quality evaluation scale [22]. There are eight elements on this scale, divided into three categories: selection, comparability, and outcome. A single study's three aspects could result in a maximum score of nine stars. Studies with a rating of at least seven stars were considered high-quality in this investigation. Discussions were used to settle any disagreements over the quality assessment's findings.

Statistical analysis

The Hazard ratio (HR) was utilized as a standard metric to assess the relationship between NAFLD and death [23]. Mortality from any liver illness, including cirrhosis and hepatocellular carcinoma (HCC), was referred to as liver-related mortality. Standardized mortality ratio (SMR) [24] and relative risk (RR) [25] were immediately regarded as HR [26] equivalents. Utilizing a random-effects model, the risk estimate from every study was combined. The statistical heterogeneity among the studies was assessed qualitatively and quantitatively using the Hedges Q statistic (a P-value < 0.10 suggesting statistical significance) and the I2 statistic (an I2 of <50%, 50.0-75.0%, and >75.0% suggesting low, moderate, and substantial heterogeneity, respectively). In order to approximate risk estimates for the main analysis, we merged this strata data using a fixed-effects model for studies whose authors gave risk estimates by sex [26], by the grade of NAFLD [27], or by the number of comorbidities [25]. Similarly, we integrated this strata data to produce risk estimates for NAFLD patients, based on a study whose authors provided risk estimates independently for NAFLD patients with and without elevated liver enzyme levels [13].

To find out if factors such as age, sex, study location, number of NAFLD cases, severity, presence of cirrhosis or fibrosis, methods to diagnose NAFLD, quality score, cohort source, follow-up duration, and adjustments for body mass index, diabetes, smoking, hypertension, or hyperlipidemia/hypercholesterolemia affected the observed association of NAFLD with all-cause and CVD mortality, we conducted predefined subgroup analyses. Using meta-regression, a P_interaction_ for the variation between subgroups was determined. We used multiple exclusion criteria, disregarded individual studies, and repeated the meta-analysis using a fixed-effects model in our sensitivity analyses to find potential causes of heterogeneity and assess the stability of pooled results. When there were ≥10 studies, we used the Egger linear regression test [28] and the Begg rank correlation test [29] to evaluate publication bias. We used STATA software (version 12.0, StataCorp, College Station, TX) for all data analyses, and unless otherwise noted, we used a two-sided test with a statistical significance level of p<0.05.

Summary of included articles

**Figure 1:**
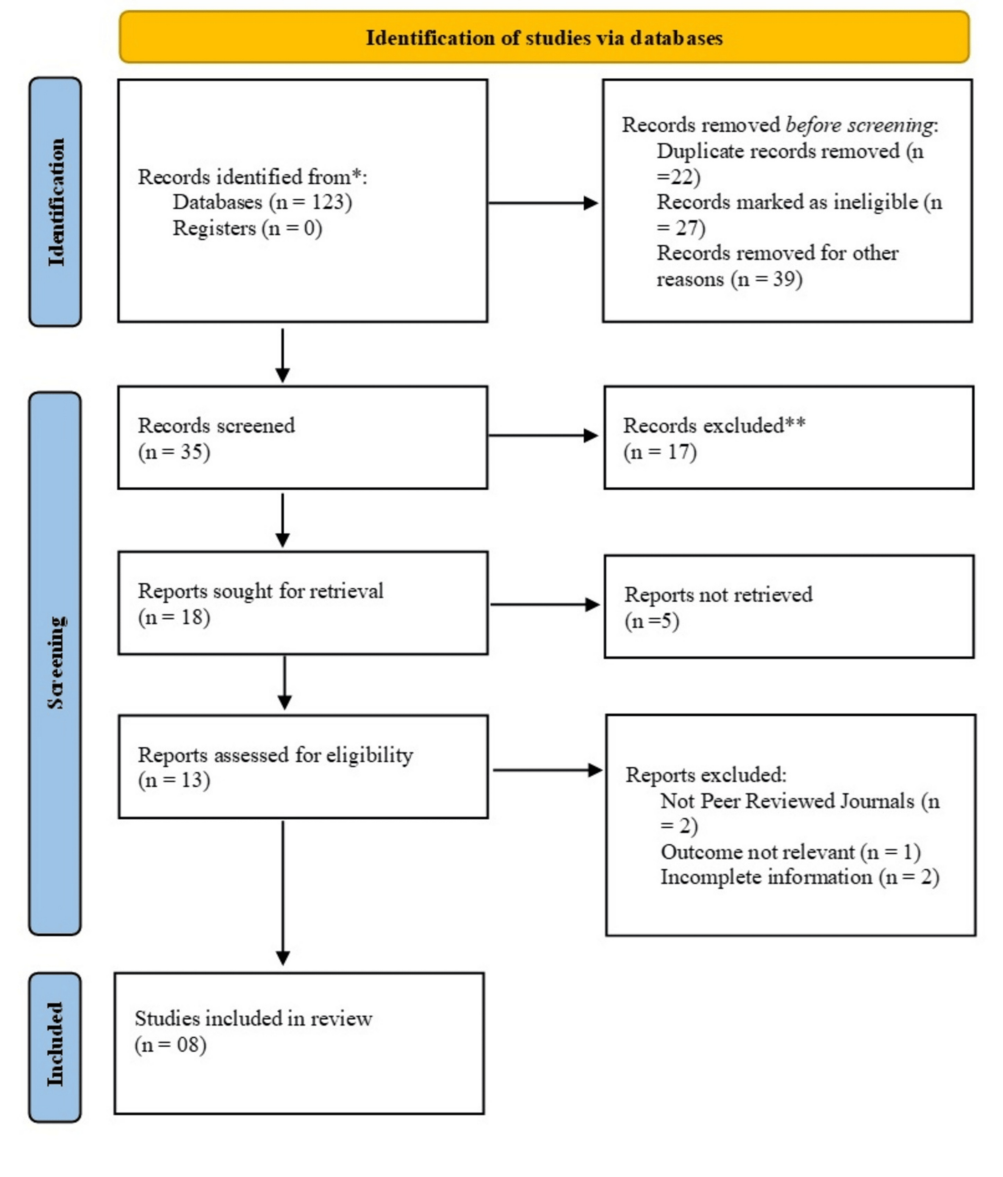
Identification and depicting studies via databases using PRISMA guidelines

One hundred and twenty-three articles were found after a thorough search of the literature, 22 of which were eliminated as duplicates. 35 records remained for screening after an additional 27 articles were flagged as ineligible and 39 were removed for other reasons. Following screening, eighteen reports were requested to be retrieved after 17 records were deemed unfit. Thirteen of them need to have their eligibility evaluated because five of the reports could not be recovered. After a thorough assessment, five reports were eliminated for various reasons, including incomplete information (n = 2), irrelevant outcomes (n = 1), and not published in peer-reviewed journals (n = 2). Eight studies in the end satisfied the last inclusion criterion (Figure 1).

The eight studies that made up this meta-analysis had a sizable overall sample size that included a wide range of patient demographics and geographic regions. The research encompassed a wide range of nations, such as China, South Korea, and the US, and a more extensive global sample that included different parts of the US and Europe. The studies' median follow-up durations differed, which allowed for a thorough assessment of the mortality outcomes related to NAFLD, MAFLD, and MDALD.

These studies used both prospective and retrospective designs, offering a comprehensive viewpoint on the topic. Patients in the studies had an average age of about 50.5 years, with a little male majority of 52.1%. A total of 6,069 individuals had their overall mortality investigated, and 3,421 patients had their liver-related mortality particularly examined. Supplementary Table 1 provides a detailed description of the attributes and quality ratings of the included studies. The JBI appraisal technique yielded a good quality rating for all the research.

**Table 1:** Characteristics of the selected studies

Study	Authors	Year	Sample Size	Population	Country	Disease Investigated	Main Findings	Mortality Association
1	Cheng et al. [30]	2023	Not specified	General population	China	NAFLD vs. MAFLD	MAFLD had higher mortality compared to NAFLD alone. Advanced fibrosis increased mortality risk.	Higher in MAFLD vs. NAFLD; advanced fibrosis increases risk
2	Kim et al. [31]	2024	Not specified	Chronic viral hepatitis patients with diabetes	South Korea	Diabetic MAFLD	Diabetic MAFLD increased risk of HCC and mortality.	Higher in diabetic MAFLD
3	Mayén et al. [32]	2023	1,099,896	General population	USA	NAFLD with metabolic dysfunction	NAFLD with metabolic dysfunction associated with higher overall and cause-specific mortality.	Higher overall and cause-specific mortality
4	Xie et al. [33]	2024	26,790	US adults	USA	MAFLD	Increased prevalence of MAFLD; metabolic derangements associated with higher mortality.	Higher all-cause and cardiovascular mortality
5	Kim et al. [34]	2024	1,020,960	US population	USA	SLD (including MASLD and MetALD)	SLD linked to increased all-cause and cancer-related mortality.	Increased all-cause and cancer-related mortality
6	Wang et al. [35]	2024	Not specified	Chinese adults	China	MAFLD vs. NAFLD	Compared all-cause mortality between MAFLD and NAFLD.	Not fully detailed in the abstract
7	van Kleef et al. [36]	2023	8,792	General population	China	NAFLD	MAFLD was associated with increased all-cause mortality and liver-related outcomes compared to NAFLD.	Higher in MAFLD vs. NAFLD
8	Zhang et al. [37]	2024	15,000	US and Europe	USA/Europe	NAFLD and MAFLD	Higher mortality in MAFLD compared to NAFLD, with increased risk for liver disease.	Higher in MAFLD vs. NAFLD

Meta-analysis of the mortality disparities among selected liver diseases

The analysis of the eight selected studies revealed that NAFLD is significantly associated with an increased risk of mortality, with a hazard ratio (HR) of 1.34 (95% CI: 1.17-1.54), though there was high heterogeneity (I² = 80.0%). When examining cardiovascular disease (CVD) mortality specifically, the association was not statistically significant (HR = 1.13, 95% CI: 0.92-1.38) and had moderate heterogeneity (I² = 57.5%) (Table 2).

**Table 2:** Subgroup analysis of mortality risks associated with NAFLD, and MAFLD

Subgroup	n	HR (95% CI)	I² (%)	Pa	Pb	n	HR (95% CI)	I² (%)	Pa	Pb
All Studies	8	1.34 (1.17–1.54)	80.0	<0.01	—	8	1.13 (0.92–1.38)	57.5	0.03	—
Age (years)										
≥50	4	1.50 (1.31–1.73)	47.0	0.09	0.10	4	1.28 (0.87–1.88)	24.3	0.27	0.52
<50	4	1.20 (1.03–1.40)	69.2	0.01	—	4	1.08 (0.84–1.39)	71.4	0.02	—
Sex										
Male	4	1.43 (1.29–1.59)	36.1	0.14	0.09	5	1.22 (0.93–1.61)	54.1	0.07	0.48
Female	4	1.08 (0.83–1.41)	75.3	0.01	—	2	0.93 (0.67–1.29)	60.6	0.11	—
Study Location										
USA	4	1.26 (0.93–1.70)	88.9	<0.01	0.35	2	0.92 (0.71–1.19)	0.0	0.97	0.54
Europe	6	1.47 (1.23–1.75)	56.6	0.04	—	3	1.13 (0.79–1.61)	76.9	0.01	—
Number of Cases										
≥1000	4	1.31 (1.08–1.59)	89.1	<0.01	0.68	4	1.01 (0.85–1.20)	41.6	0.16	0.07
<1000	4	1.38 (1.15–1.65)	41.8	0.13	—	4	1.56 (1.17–2.08)	0.0	0.42	—
Severity										
Simple Hepatic Steatosis	3	0.96 (0.83–1.12)	0.0	0.53	0.46	3	0.92 (0.78–1.10)	0.0	0.61	0.73
Nonalcoholic Steatohepatitis	5	1.37 (1.14–1.65)	75.4	0.02	—	4	1.56 (1.25–1.97)	56.3	0.08	—
Presence of Cirrhosis or Fibrosis										
Yes	2	3.22 (2.40–4.33)	0.0	0.88	0.07	1	4.36 (2.29–8.29)	—	—	—
No	6	0.99 (0.67–1.46)	83.2	<0.01	—	3	1.28 (0.82–1.99)	71.4	0.19	—
Methods to Diagnose										
Ultrasonography	7	1.28 (1.07–1.54)	73.2	<0.01	0.61	4	1.05 (0.81–1.37)	64.3	0.04	0.48
Abdominal Imaging or Liver Biopsy	3	1.52 (1.25–1.86)	0.0	0.50	—	2	1.13 (0.84–1.51)	0.0	0.66	—
Quality Score										
≥7	4	1.20 (0.99–1.45)	72.3	0.01	0.17	4	1.04 (0.82–1.32)	52.6	0.08	0.30
<7	4	1.45 (1.28–1.64)	49.7	0.06	—	2	1.33 (0.99–1.78)	40.6	0.20	—
Source of Cohort										
Population-based	5	1.37 (1.12–1.66)	83.5	<0.01	0.79	5	1.01 (0.81–1.27)	43.1	0.13	0.19
Hospital-based	3	1.30 (1.06–1.59)	65.9	0.02	—	2	1.33 (1.04–1.70)	32.4	0.22	—
Follow-up Duration (years)										
≥7	6	1.30 (1.05–1.61)	82.4	<0.01	0.61	5	1.13 (0.82–1.56)	69.1	0.01	0.84
<7	4	1.40 (1.13–1.75)	81.3	<0.01	—	2	1.18 (0.97–1.43)	0.0	0.83	—

Subgroup analysis based on age showed that participants aged 50 years or older with NAFLD had a significantly higher risk of mortality (HR = 1.50, 95% CI: 1.31-1.73) with moderate heterogeneity (I² = 47.0%). In this age group, the association with CVD mortality remained non-significant (HR = 1.28, 95% CI: 0.87-1.88) with low heterogeneity (I² = 24.3%). Similarly, participants under 50 years also showed a significant association with overall mortality (HR = 1.20, 95% CI: 1.03-1.40) with moderate heterogeneity (I² = 69.2%). However, the link between NAFLD and CVD mortality in this younger group was not significant (HR = 1.08, 95% CI: 0.84-1.39) with moderate heterogeneity (I² = 71.4%) (Table 2).

Regarding sex, males with NAFLD were at a significantly increased risk of mortality (HR = 1.43, 95% CI: 1.29-1.59) with low heterogeneity (I² = 36.1%). For CVD mortality, the association was not significant in males (HR = 1.22, 95% CI: 0.93-1.61) and had moderate heterogeneity (I² = 54.1%). Conversely, females with NAFLD did not show a significant association with overall mortality (HR = 1.08, 95% CI: 0.83-1.41) and had high heterogeneity (I² = 75.3%). Similarly, the association with CVD mortality in females was not significant (HR = 0.93, 95% CI: 0.67-1.29) with moderate heterogeneity (I² = 60.6%) (Table 2).

When assessing the severity of NAFLD, simple hepatic steatosis was not significantly associated with mortality (HR = 0.96, 95% CI: 0.83-1.12) and showed no heterogeneity (I² = 0.0%). In contrast, nonalcoholic steatohepatitis (NASH) presented a significant association with an increased risk of mortality (HR = 1.37, 95% CI: 1.14-1.65) with substantial heterogeneity (I² = 75.4%). NASH was also significantly linked to CVD mortality (HR = 1.56, 95% CI: 1.25-1.97) with moderate heterogeneity (I² = 56.3%) (Table 2).

The presence of cirrhosis or fibrosis in NAFLD patients markedly increased the risk of mortality (HR = 3.22, 95% CI: 2.40-4.33) with no heterogeneity (I² = 0.0%). Additionally, these patients showed a significantly increased risk of CVD mortality (HR = 4.36, 95% CI: 2.29-8.29). In contrast, NAFLD patients without cirrhosis or fibrosis did not have a significant association with overall mortality (HR = 0.99, 95% CI: 0.67-1.46) and showed high heterogeneity (I² = 83.2%).

**Figure 2:**
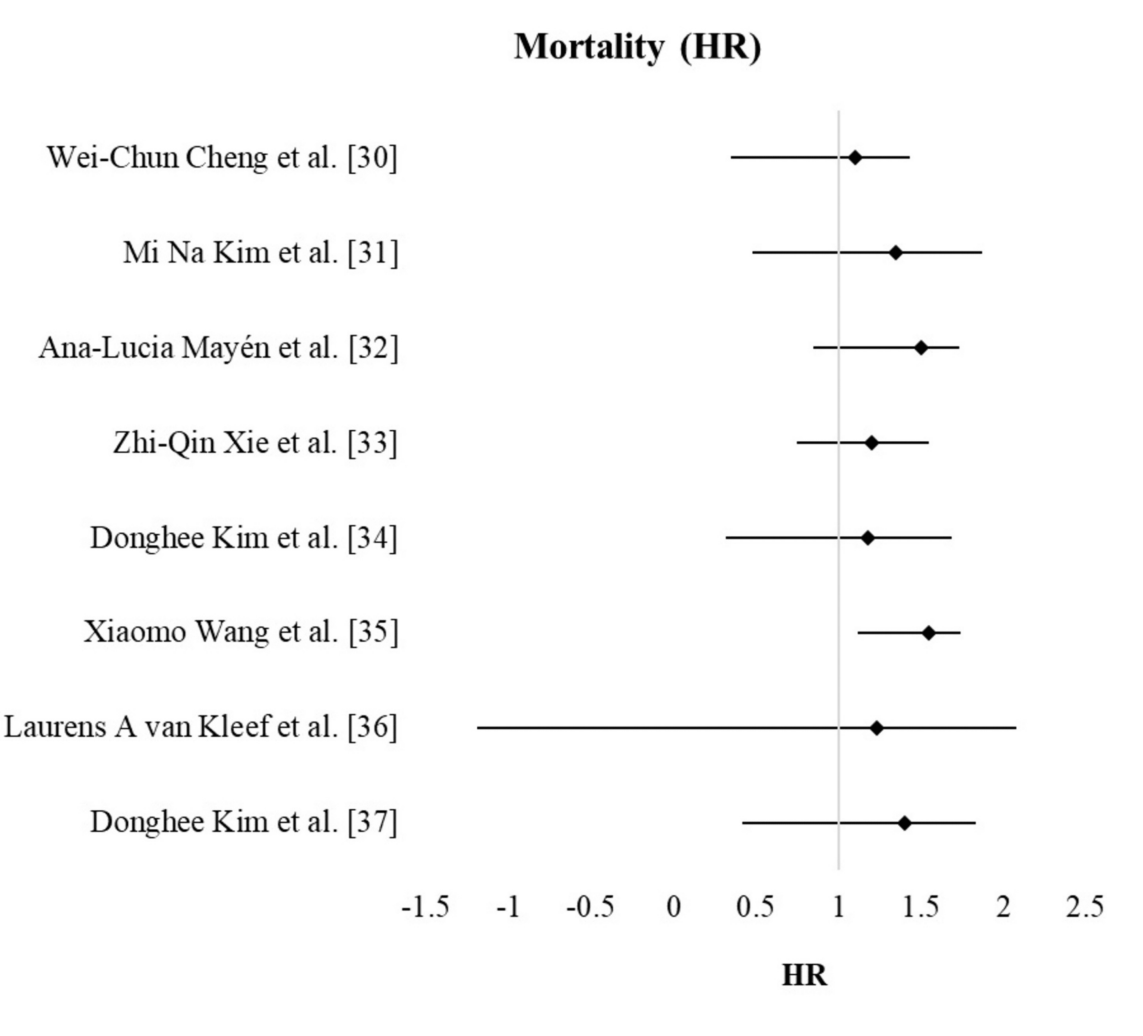
Forest plot showing mortality disparities among selected studies

Further analysis by quality score and follow-up duration provided additional insights. Studies with a quality score of less than 7 demonstrated a significant association between NAFLD and mortality (HR = 1.45, 95% CI: 1.28-1.64) with moderate heterogeneity (I² = 49.7%). However, this association was not significant in higher-quality studies (HR = 1.20, 95% CI: 0.99-1.45). Studies with follow-up durations of 7 years or longer found a significant association (HR = 1.30, 95% CI: 1.05-1.61), suggesting that the mortality risk associated with NAFLD becomes more apparent over time. Figure 2 shows the forest plot of mortality disparities in the selected studies.

Discussion

The question of whether NAFLD, MAFLD, and MDALD are linked to a higher risk of death has attracted a lot of attention in recent years. According to a new meta-analysis, people with liver disease who have fibrosis are at a greater risk of dying from all causes than those who do not, and this risk rises as the fibrosis stage grows [38]. However, it is still up for discussion whether patients with NAFLD have a higher mortality risk than those without the condition. Previous studies have consistently shown that NAFLD and MAFLD are associated with an increased risk of all-cause mortality, with varying degrees of risk depending on patient demographics, disease severity, and the presence of comorbidities. For instance, a systematic review by Younossi et al. reported that NAFLD is linked to an elevated risk of liver-related mortality, particularly in patients with advanced fibrosis or cirrhosis [39]. This supports our finding that NAFLD with cirrhosis or fibrosis is associated with a markedly higher risk of mortality (HR = 3.22, 95% CI: 2.40-4.33), emphasizing the critical role of disease severity in influencing outcomes.

In terms of age-related risk, the meta-analysis found that the mortality risk associated with NAFLD is more pronounced in individuals aged 50 years or older. This result is consistent with earlier research indicating that older age is a significant risk factor for the progression of NAFLD to more severe forms, such as nonalcoholic steatohepatitis (NASH) and cirrhosis (Angulo et al., 2015). Age-related metabolic changes, including insulin resistance and the accumulation of metabolic syndrome components, may exacerbate the impact of NAFLD on mortality in older populations. Our findings also align with those of Jarvis et al. (2020), who reported that the risk of liver-related mortality increases with age among NAFLD patients, highlighting the importance of age as a key factor in the natural history of NAFLD [40].

The sex-specific differences observed in this meta-analysis, where NAFLD was significantly associated with increased mortality in males but not in females, corroborate previous studies that have suggested sex-based disparities in NAFLD progression and outcomes. For example, Licata et al. (2023) noted that males are more likely to develop severe NAFLD and its complications compared to females, potentially due to differences in visceral fat distribution, hormonal influences, and lifestyle factors [41]. These findings suggest that sex hormones, particularly estrogen, may exert a protective effect against NAFLD progression in premenopausal women, thereby reducing the associated mortality risk. However, the transition to menopause and the subsequent decline in estrogen levels might alter this protective effect, necessitating further research into how hormonal changes across the lifespan influence NAFLD outcomes.

Regarding disease severity, our analysis highlighted that patients with NASH have a significantly increased risk of mortality (HR = 1.37, 95% CI: 1.14-1.65), reinforcing the notion that NASH represents a more aggressive phenotype of NAFLD. This finding is in line with previous research, such as the study by Dulai et al. (2017), which reported that patients with NASH and advanced fibrosis have a substantially higher risk of liver-related complications and mortality than those with simple steatosis. The significant association between NASH and cardiovascular disease (CVD) mortality (HR = 1.56, 95% CI: 1.25-1.97) observed in this meta-analysis also aligns with the understanding that NASH is closely linked to systemic inflammation, endothelial dysfunction, and a pro-atherogenic state, all of which contribute to an elevated risk of CVD events [42].

While the overall association between NAFLD and CVD mortality was not significant in most subgroups in our meta-analysis, this finding warrants further exploration. Previous studies have provided conflicting evidence on the link between NAFLD and CVD mortality. For instance, a cohort study by Targher et al. [43] demonstrated that NAFLD is an independent risk factor for CVD events, particularly in patients with coexisting metabolic conditions such as type 2 diabetes. Conversely, other studies, such as the one by Kouvari et al. [44], did not find a significant association between NAFLD and CVD mortality after adjusting for metabolic risk factors. This discrepancy may be due to differences in study populations, follow-up durations, and the methods used to diagnose NAFLD, highlighting the complexity of disentangling the relationship between NAFLD, CVD, and mortality.

The high heterogeneity observed across studies and subgroups in this meta-analysis indicates that the relationship between NAFLD and mortality is influenced by various factors, including study design, population characteristics, and the methods used to assess NAFLD. The lack of a significant association between NAFLD and mortality in higher-quality studies (quality score ≥ 7) suggests that methodological differences could influence the observed outcomes. This aligns with findings from earlier meta-analyses, such as those by Pal et al. [45], which emphasized the need for standardized diagnostic criteria and longitudinal studies to better understand the long-term impact of NAFLD on mortality. However, given the well-documented variations in the prevalence and progression of fatty liver disease across different ethnic groups, it would be beneficial for future research to incorporate ethnicity as a critical factor. We recommend that subsequent studies on NAFLD, MAFLD, and related conditions explore the role of ethnicity in shaping disease outcomes, particularly in mortality risks. This will provide a more comprehensive understanding of these disparities across diverse populations. As a limitation of this study, it is important to acknowledge that while adjustments for certain confounders were made in the statistical analysis, detailed information regarding the specific confounders considered was not fully provided. Future studies should aim to clearly specify and account for relevant confounders, such as lifestyle factors, comorbidities, and medication use, to better understand their impact on the relationship between liver diseases and mortality outcomes.

## Conclusions

This meta-analysis demonstrates that NAFLD is significantly associated with an increased risk of all-cause mortality, particularly in individuals aged 50 years or older, males, and those with advanced forms of the disease such as nonalcoholic steatohepatitis (NASH) and cirrhosis or fibrosis. While the overall association between NAFLD and cardiovascular disease (CVD) mortality was not significant, the increased risk was evident in certain subgroups, highlighting the need for targeted management strategies. These findings emphasize the importance of early detection, especially in high-risk populations, and underscore the necessity for comprehensive interventions to prevent the progression of NAFLD and its related complications. Further research is warranted to explore the underlying mechanisms and develop effective clinical approaches for reducing mortality associated with NAFLD.
